# Caught between Two Cultures: Understanding Mental Health and Help-Seeking among Australian Indian Families

**DOI:** 10.1007/s10903-025-01777-9

**Published:** 2025-09-17

**Authors:** Bindu Joseph, Robeena Emmanuel, Michael Olasoji

**Affiliations:** Federation University, Ballarat, Australia

**Keywords:** Migrant Populations, Mental Health, Mental Health Awareness, Culturally and Linguistically Diverse (CALD), Indians

## Abstract

Mental illness often emerges before the age of 25, with suicide being the leading cause of death among young people in Australia. Migrant communities, including the Indian-born population, experience high rates of mental illness and delayed help-seeking. Parents and caregivers in these communities play a key role in supporting their children’s mental health. The study aimed to examine the mental health awareness and help-seeking behaviour of Indian adults in Australia and their impact on their children from the perspectives of parents. Semi-structured interviews were conducted with 14 Indian migrant parents and caregivers, and data were analysed using thematic analysis. Findings revealed themes related to dual cultural challenges, limited understanding of mental health, and barriers to seeking help due to cultural stigma and unfamiliarity with the Australian system. Community support and tailored resources were identified as enablers, although access to these resources remained a barrier. Culturally appropriate mental health education and resources are vital to improving mental health outcomes for Indian migrant families.

## Background

 Mental health awareness refers to individuals’ understanding, beliefs, and practices regarding mental health conditions, their causes, and the pathways to seeking help [18]. This concept is particularly crucial for migrant populations, where cultural, social, and systemic factors can significantly influence mental health outcomes [10, 44]. Moving to another country brings cultural unfamiliarity and a loss of social connections and support systems [26, 32, 36]. Cultural diversity worldwide has multidimensional impacts on mental health, from how health and illness are perceived to help-seeking behaviours and overall attitudes [15].

The 2021 census reveals that 976,000 people in Australia have Indian ancestry [1]. Furthermore, among these migrants, only 1.5 per cent self-identified as having any mental health conditions [1]. In the cultural context of India, interdependence with family members and collectivism are significant aspects of life [19, 47]. India-born people may associate mental illness with severe stigma and shame, or denial may lead to reluctance to access mental health services [8, 24, 30, 46, 50]. Due to negative attitudes towards mental illness, seeking help for mental health problems usually only occurs in severe cases and may start with the pursuit of traditional treatment options [8, 48]. Research also highlights that separation from the interdependent family system can impact emotional well-being [11]. It is assumed that multiple aspects of migrant experiences impact mental health and access to support services.

Many Indian migrants may not be fluent in English, making it challenging to understand mental health resources or articulate their needs effectively [5, 28]. Furthermore, a possible gap in health literacy and the difficulty in finding appropriate terms and phrases to express symptoms can further complicate this issue [16, 27, 29, 48, 50, 51]. This communication gap may lead to feelings of inadequacy and negatively impact mental health and well-being.

Research suggests that in the host country, navigating mental health services or understanding what constitutes appropriate care can be challenging for migrants [12, 20]. This unfamiliarity and stigma can delay help-seeking and contribute to the chronicity and deterioration of symptoms [2, 24, 30]. The unfamiliarity and lack of culturally appropriate care can also lead to general distrust towards formal mental health systems [14, 16, 21]. This distrust could be rooted in previous experiences or cultural beliefs about health and illness [2]. Furthermore, family members, including parents and caregivers, who provide emotional support to young people play a vital role in identifying mental health issues and accessing and engaging with mental health services [22, 48, 50, 51].

In Australia, more than one-fifth of children (23% or around 995,700) aged 0–14 had both parents born overseas. Two-thirds (66%) were born in mainly non-English-speaking countries; the largest groups were from India (11%), China (6%), and the Philippines (5%) [41]. A range of factors can influence the mental health of children, including exposure to events prior to migration and post-migratory stressors, such as settling into a new country and culture [37, 41]. This makes the mental health needs and issues of migrant children distinct from those of their domestic counterparts [40]. A study on second-generation migrants in Turkey concluded that higher rates of major mental illnesses, including depression, post-traumatic stress disorders and anxiety disorders, are found among second-generation migrants [42]. Second-generation migrants are individuals born in the host country to at least one parent who was born in a different country and migrated to the host country. Previous studies on the mental health of second-generation migrant children are rare. Adverse experiences and traumatic life events of migration influence children. Studies indicated that second-generation immigrant children’s / young people’s mental health problems are mostly related to intergenerational conflicts caused by asymmetric acculturation within the family, lack of adequate support from their parents, and restrictive processes due to discrimination [39, 45]. Similarly, another study in Europe also highlights the high risk of suicide among the second-generation migrants due to intergenerational acculturation conflict [38].

Parents play a significant role in accessing psychological services for children/young people, often acting as the primary decision-makers and initiators of help-seeking. However, research has shown that many parents lack the necessary knowledge and skills to recognise and manage their child’s mental health concerns effectively [49]. This gap likely contributes to the ongoing disparity in children receiving appropriate mental health treatment [28, 31, 48]. Other studies have also highlighted that parents’ mental health awareness is strongly linked to the utilisation of mental health services and help-seeking for children [50, 51].

Given these complexities, it is essential to explore the factors influencing the mental health awareness and help-seeking of Indian migrants. The study aimed to examine the mental health awareness and help-seeking behaviour of Indian adults in Australia and their impact on their children from the perspectives of parents.

## Theoretical/conceptual Framework

**Fig. 1:**
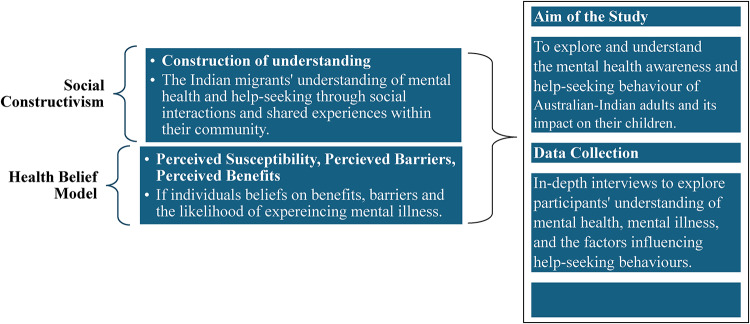
Conceptual Framework

The study employed a qualitative, exploratory, and descriptive approach, which allows (Figure 1) for exploring, describing, and understanding a phenomenon of interest that extends beyond mere observation [17]. This design is beneficial when there is a gap in the available literature, as it helps uncover new knowledge and gain an understanding of the meanings of the phenomena under study [9].

This study draws upon theoretical perspectives of ‘social constructivism’ and the ‘Health Belief Model’ to explore mental health awareness and help-seeking behaviours among Australian Indian adults, as well as how these behaviours may impact their children [43, 46]. The social constructivism theory suggests that individuals’ understanding of concepts such as mental health and illness is shaped through cultural, social, and communal interactions [33]. Within this framework, the meanings attached to mental health are unique to the individual and constructed through everyday conversations, traditions, and shared experiences. For Indian migrants, these understandings are influenced by both their cultural beliefs and practices, as well as the sociocultural context of Australia. By applying this theory, the study examines how Indian adults in Australia perceive mental health and the process of seeking help. The participants’ perspectives are shaped not only by their individual experiences but also by collective norms, community narratives, and social expectations.

The Health Belief Model (HBM) helps to explain health-related behaviours by focusing on individuals’ beliefs about health conditions [43]. In this study, three components of the HBM are relevant: Perceived Susceptibility (the individual’s belief about the likelihood of experiencing mental health issues), Perceived Barriers (the perceived obstacles to seeking help), and Perceived Benefits (the perceived effectiveness or value of seeking help). Applying these elements of the HBM allows for a deeper understanding of the decision-making process that Indian adults engage in when considering whether to seek help for mental health concerns. These beliefs and behaviours might influence parents’ and children’s beliefs, openness to mental health discussion, and future help-seeking attitudes.

## Methods

This study was approved by the human research ethics committee of the University (reference 2023/110).

### Participants

Participants (*n* = 14) were recruited using purposive sampling, which enabled the intentional selection of individuals likely to provide rich, relevant, and diverse insights into the research topic. This approach is beneficial in qualitative research for capturing depth and variation in participants’ experiences [9].

Recruitment was conducted through advertisements distributed via various Indian cultural organisations and community groups across Australia. The inclusion criteria required participants to be:

- Adults of Indian origin currently living in Australia, and.
- Either parents, caregivers of children, or second-generation migrants.

Indian migrants who did not have children or caregiving responsibilities were excluded from the study.

### Data Collection

Data were collected through semi-structured, in-depth interviews with the 14 participants meeting the inclusion criteria. All participants were provided informed consent before the interview. Interviews lasted between 45 min and one hour and were conducted either face-to-face or online, depending on the participant’s preferences. A set of open-ended probes designed to explore migrant parents’ perceptions and experiences related to mental health and mental health services in Australia guided interviews. They covered key areas such as participants’ migration background, understanding of mental health, perceptions of their children’s wellbeing, experiences with mental health services, cultural attitudes and stigma, barriers and facilitators to accessing care, and suggestions for improving mental health support (please see Appendix 1).

The research team consisted of three academic researchers with expertise in qualitative methodologies and content analysis. All team members had Culturally and Linguistically Diverse backgrounds and substantial experience in conducting culturally sensitive interviews. The team’s cultural competence supported rapport-building and enhanced the credibility of data collection. Throughout the study, the research team engaged in ongoing reflexive practice to critically examine how their own cultural identities, personal experiences, and assumptions might influence the data collection and interpretation process. Reflexive journaling, peer debriefing, and regular discussions were used to surface potential biases and ensure interpretations remained grounded in participants’ narratives, rather than researchers’ perspectives. Data saturation was reached after the twelfth interview, with no new themes emerging. Two additional interviews were conducted to confirm thematic consistency and ensure depth of understanding.

### Data Analysis

Thematic analysis was conducted using the six-step reflexive thematic analysis approach outlined by Braun and Clarke [7]. This included familiarisation with the data, generation of initial codes, searching for themes, reviewing themes, defining and naming themes and producing the report (Table 1).

Analysis was also guided by a social constructivist lens, focusing on how participants made sense of mental health and help-seeking behaviours within their social and cultural contexts. The coding process was inductive, allowing patterns to emerge naturally from the data. Themes were developed through a process of constant comparison across interviews and multiple rounds of analysis. Regular research team discussions allowed reflexivity and ensured alignment with the study’s theoretical framework. These meetings also facilitated consistency and transparency in theme development, enhancing the trustworthiness of the findings (Table 2).

**Table 1:**
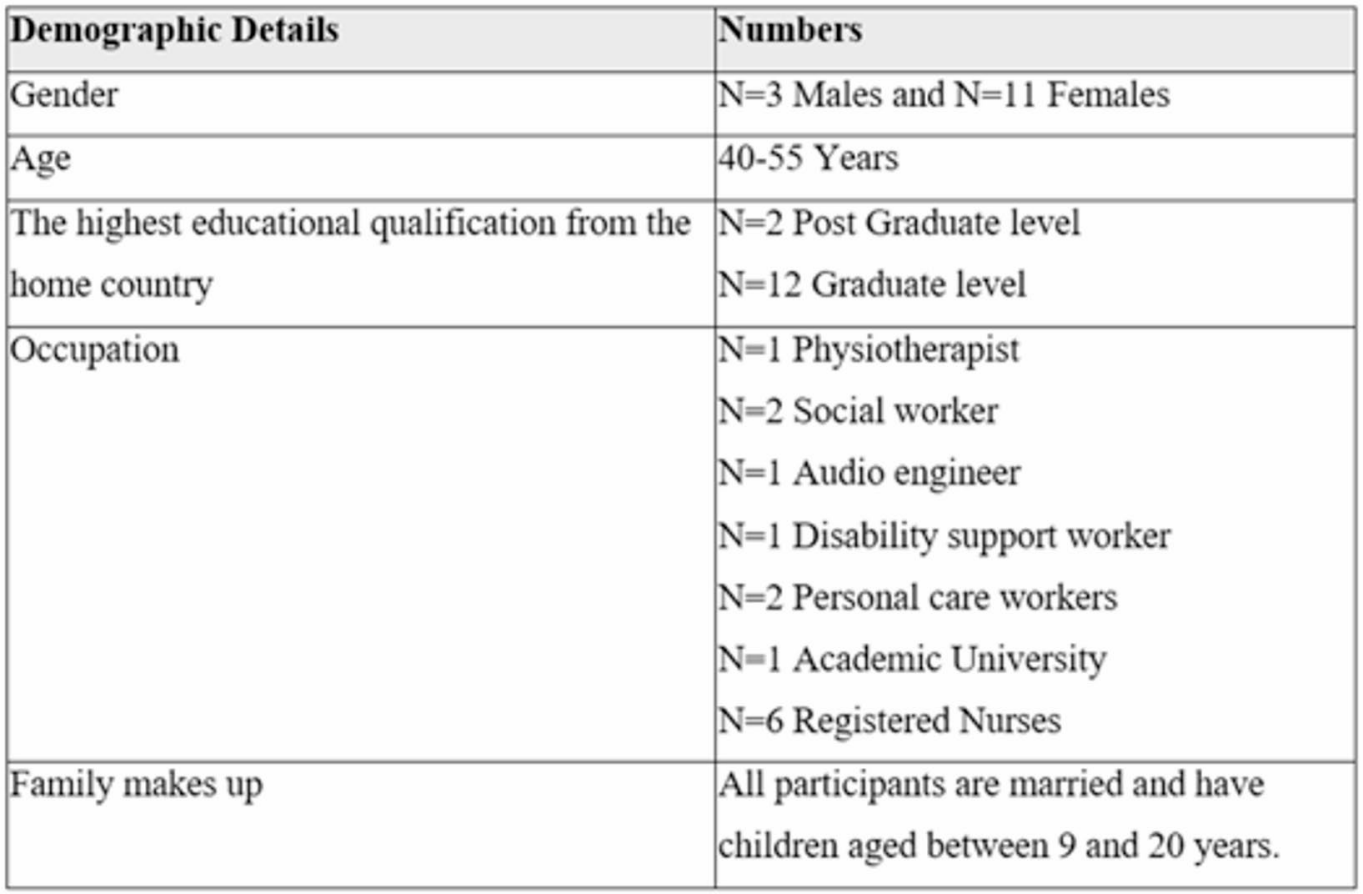
Participant’s profile

## Results

Fourteen participants (3 males and 11 females) were included in this study. All the participants had children between 9 and 20 years old. All participants were migrants from India. The average post-migration time was 8 years.

**Table 2:**
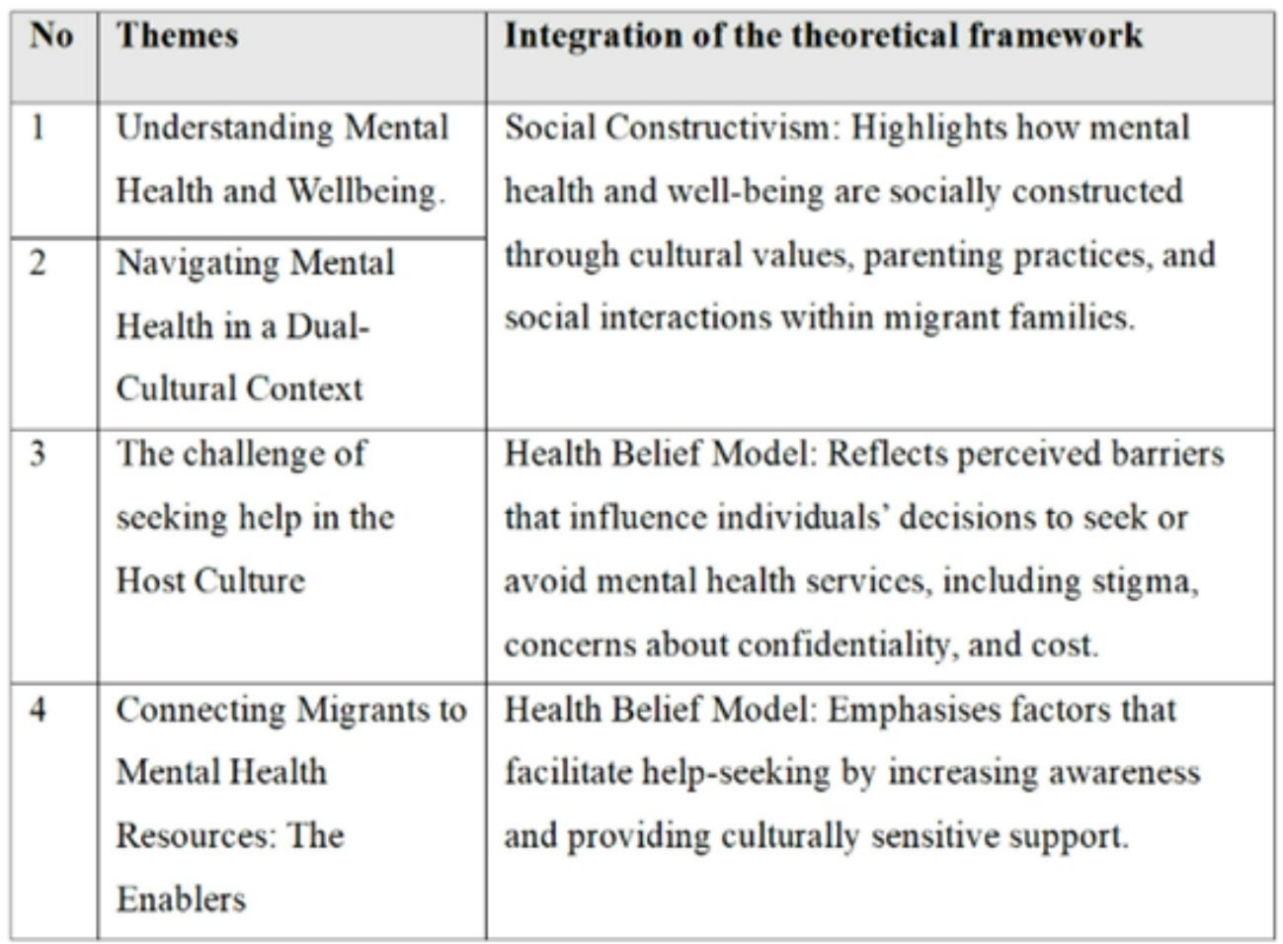
Themes

Thematic analysis of the interviews revealed four major themes: (1) Understanding Mental Health and Well-being, (2) Navigating Mental Health in a Dual Cultural Context, (3) The Challenge of Seeking Help in a New Culture, and (4) Connecting Migrants to Mental Health Resources: The Enablers. These themes highlight how Indian migrant parents and caregivers conceptualise mental health, experience intergenerational and cultural conflict, and identify both the barriers and facilitators to seeking mental health support in the Australian context.

### Theme 1: Understanding Mental Health and Well-being

Participants’ understanding of mental health centred around the concepts of being happy, relaxed, socially active and having a good relationship with others. Mental health wellbeing was often associated with being emotionally content, stress-free, and socially engaged. Several participants described good mental health in terms of experiencing positive emotions and having a harmonious family life.

“Being happy and having a peaceful life with little stress means feeling relaxed, spending time with the family, and having meals with the family.” (P10).

Mental health was also conceptualised through observable behaviours, such as smiling, socialising, and remaining active. Participants frequently described those with “good” mental health as appearing outwardly happy and engaged with their surroundings.

“They look happy, in a happy mood with happy faces, engage in their activities, and perform what they are meant to perform properly and communicate with others.” (P13).

Social connection was seen as essential to well-being. Participants emphasised the importance of maintaining strong ties with family, friends, and community groups, noting that isolation negatively affected both adult and child mental health.

“I would say they need to have a good, positive relationship with their family and friends… having some good positive connections.” (P1).

This theme highlights that participants’ mental health and wellbeing concepts were often shaped by cultural norms that prioritise emotions, visible social functioning, and collective well-being over clinical or psychological definitions of mental health.

### Theme 2: Navigating Mental Health in a Dual Cultural Context

Parenting in a bicultural environment caused emotional and relational challenges for participants. Many described ongoing tensions with their children due to differing cultural expectations, often leading to misunderstandings and intergenerational conflict. These struggles affected both parents’ and children’s emotional well-being.

“My child seems caught between two worlds.” (P3).

“As parents, we follow certain cultural practices at home, and my teenage children question them.” (P6).

Children’s questioning of traditional values, especially during adolescence, often created stress for parents, who struggled to reconcile Indian cultural practices with their children’s upbringing in Australia. This disconnect was seen as a source of mental strain for both generations.

“Cultural diversity is affecting our kids’ mental health… I’m confused about whether I am right or they are right. You know, like that’s a conflict of matching concepts and culture that’s affecting the mental health that’s struggling” (P10).

Compounding these challenges was the absence of extended family support. Participants frequently discussed how parenting without grandparents or relatives created additional emotional and logistical burdens, both for themselves and for their children. The lack of familial networks contributed to feelings of isolation and concern about their children’s social development.

“We’re far from our extended family, and it’s hard for all of us. I worry about how this separation affects my child and how they miss their grandparents and cousins.” (P3).

This theme reflects how parents navigate cultural duality, often without sufficient support, while trying to maintain family cohesion and mental well-being.

### Theme 3: the Challenge of Seeking Help in the Host Culture

Participants identified multiple barriers to seeking mental health support, with stigma, privacy concerns, and cost emerging as the most significant. Mental health issues were often perceived as shameful within their cultural communities. Most of the participants expressed the impact of the collectivistic nature of Indian culture on stigma and its interference with help-seeking.

“You know that kind of issue in my community… this is a shame for our family… I am scared.” (P6).

Participants feared social judgment and potential exclusion from their community, especially in relation to future marriage prospects or family reputation.

“You will not be married to another family when you’re having these mental health issues… that is a significant stigma.” (P11).

Concerns about privacy further discouraged help-seeking. Many participants felt that in small, tightly knit communities, mental health problems could not be kept confidential and might result in gossip or reputational damage.

“I worry that if anyone from my community sees me in the mental health clinic, they’ll think I have problems; they’ll talk about me.” (P3).

Financial burden was another commonly reported barrier. Even with Medicare rebates, participants expressed difficulty affording regular therapy or psychological services.

“It’s not free and very expensive. I paid $160 per session after the GP (General Practitioner) rebate, but I couldn’t afford it.” (P3).

These findings highlight the interplay of cultural stigma, fear of exposure, and economic challenges that restrict access to timely and appropriate mental health support.

### Theme 4: Connecting Migrants To Mental Health Resources: the Enablers

Despite barriers, participants identified practical and culturally relevant enablers that could improve mental health awareness and service use. Targeted community-based education and awareness programs delivered in native languages and trusted settings were also suggested.

“Education sessions during community gatherings… awareness sessions in our language, like within that community.” (P13).

Participants suggested that GPs, community leaders, schools, and religious groups could play a crucial role in normalising mental health discussions and providing guidance on accessing care.

“The government has to arrange education for Indians because they see mental health problems very negatively.” (P14).

The importance of culturally sensitive service delivery was also widely emphasised. Participants reported that clinicians’ lack of cultural understanding sometimes led to mistrust or communication breakdowns. They called for providers who could recognise culturally specific expressions of distress and build rapport with migrant families.

“Sometimes psychology people don’t understand our culture, and some things are everyday in our culture, which might not be appropriate in Aussie culture.” (P11).

Participants believed that when services were tailored to their cultural needs, both in content and approach, they would feel more confident accessing support for themselves and their children.

## Discussion

The findings of this study revealed that Indian parents in Australia often conceptualise mental health through an emotional and social lens. Being happy, relaxed, socially active and maintaining good relationships were commonly described as signs of good mental health. This understanding aligns with the World Health Organisation’s definition of mental health as “a state of well-being in which individuals can cope with daily stress, function productively, and contribute to their communities” [34, 35]. As in other research, these indicators reflect a culturally shaped view of mental health that prioritises emotional stability and visible social functioning [13]. Many parents viewed strong interpersonal connections with family and community as essential to well-being.

However, while participants demonstrated a general awareness of what constitutes positive mental health, they expressed uncertainty about recognising mental illness, understanding its symptoms, and determining when to seek help. This echoes findings from a Canadian study, where Asian parents were shown to interpret their children’s emotional or behavioural symptoms through the lens of cultural expectations, often delaying formal help-seeking [23, 44]. The influence of parental expectations and cultural values plays a significant role in shaping mental health understanding and behaviour within Indian migrant families.

Parenting in a dual cultural environment was described as emotionally and relationally challenging. Participants reported frequent tensions with their children due to differences in cultural expectations, which often led to intergenerational conflict and emotional distress. The findings of this study support the existing literature, which identifies such conflict as a common experience for migrant families navigating two cultural frameworks [46]. Second-generation migrants were described as adapting more rapidly to Australian cultural norms, often questioning traditional values and behaviours upheld by their parents. This dynamic contributed to confusion, stress, and identity struggles among children. Previous studies have documented that second-generation migrants face challenges related to discrimination, identity, and acculturation that can adversely affect their mental health [39]. Literature also suggests that differences in acculturation levels and language use can increase the likelihood of intergenerational conflicts [47].

Participants also discussed the emotional and practical challenges of raising children without the support of their extended family. In Indian collectivist culture, the extended family often plays a vital caregiving role [39], and the absence of such support in Australia is seen to impact both parent and child well-being negatively. This is consistent with findings that the loss of kinship networks can have detrimental effects on the mental health of migrants [26]. The current study highlights how culturally constructed parenting values may conflict with host country norms, contributing to identity tension, familial stress, and broader mental health challenges for both generations. The role of cultural expectations was also significant. As previous research notes, parents in Asian communities often interpret mental health symptoms based on cultural norms, which can delay or prevent professional intervention [44]. The present study confirms that these norms exist among Indian families in Australia.

Participants identified multiple barriers to accessing mental health services, including stigma, privacy concerns, and financial limitations. Cultural stigma was one of the most frequently cited issues. Many participants feared social judgment from within their community. They expressed concern about how mental illness might affect their family’s reputation and their children’s future, particularly about marriage prospects. These concerns reflect findings in the literature, which show that stigma related to mental illness is prevalent in Indian society and significantly discourages help-seeking behaviour [24, 30, 46, 50]. Stigma also contributes to fears around visibility and confidentiality. Participants worried that others in their community might see them accessing mental health services, and that it would cause reputational damage. Similar issues were reported in prior studies, where members of CALD communities avoided interpreter services due to concerns about privacy [3, 4, 31, 32].

Financial cost was another significant barrier. While a few participants expressed a willingness to seek private services, they were discouraged by the expense, even after Medicare rebates. One participant reported paying $160 per session despite using a General Practitioner referral. Others expressed uncertainty about how to access subsidised services, and no participants mentioned public mental health systems, despite being educated to at least a graduate level. Literature also indicated that private mental health services in Australia are often financially out of reach for many migrants [20].

Despite the challenges, participants identified several culturally appropriate strategies to improve mental health awareness and support within their community. Most notably, 86% of participants believed that targeted mental health awareness programs would benefit the Indian migrant population. They emphasised the importance of delivering these programs in native languages and within trusted settings such as religious or community organisations. Participants suggested that collaboration with religious leaders and cultural groups could help reduce stigma and normalise discussions around mental health. This aligns with findings that family, community, and religious networks play a central role in the mental health support of CALD populations [21, 48, 51]. Training frontline service providers in the multicultural sector, including those in settlement services and religious institutions, was seen as a potential facilitator for improved access [25]. Additionally, participants emphasised the need for culturally sensitive service delivery. Several shared that mainstream mental health professionals lacked understanding of Indian cultural norms, which led to mistrust and communication barriers. Consistent with previous research [14, 20, 21], participants believed that culturally tailored services could help foster open communication and improve comfort with seeking help.

Interestingly, although some participants were aware of GP Mental Health Care Plans and employee assistance programs, none mentioned public mental health services. This gap may be due to either a lack of knowledge or ongoing cultural reluctance to discuss such issues openly.

### New Contribution To the Literature

This study reinforces previous research on the mental health awareness and help-seeking behaviours of culturally and linguistically diverse (CALD) communities, with a specific focus on the Indian migrant population in Australia. Participants in this study highlighted how dual cultural parenting, stigma around mental illness, concerns about privacy, and financial constraints influence mental health support-seeking. Stigma and shame were reported as significant barriers, particularly due to fear of judgment from within closely knitted community networks. Concerns about confidentiality, especially around community members discovering mental health concerns, further discouraged help-seeking. These challenges have been well-documented across CALD groups and are consistent with previous findings.

While the themes identified in this study are not new, the research adds value by contextualising these experiences specifically within the Indian migrant community in Australia. Participants emphasised the importance of culturally appropriate awareness programs and the need for services to be delivered in trusted environments such as religious or cultural organisations. These suggestions align with earlier research that recommends culturally sensitive and community-based approaches to enhance mental health literacy and access among CALD populations.

### Implications

The findings highlight the need for increased access to culturally appropriate, affordable, and flexible mental health services for Indian migrant families in Australia. Participants indicated that support delivered through familiar settings, such as community groups or religious organisations, would reduce stigma and improve engagement. There is also a need for targeted mental health education within these communities, particularly focused on navigating the Australian healthcare system. Some participants were unaware of existing services, such as the GP Mental Health Plan, indicating a gap in service knowledge even among highly educated individuals. Partnerships between mainstream mental health providers and immigrant-serving agencies may increase trust and improve access. The findings of this study are consistent with Social Constructivist theory, which highlights how individuals’ understanding of mental health is shaped through social interaction, culture, and experience. Parents’ perceptions and behaviours are constructed through the lens of Indian cultural norms, social expectations, and their lived experience as migrants. These constructed beliefs influence how they interpret their children’s mental health, their comfort with seeking support, and their attitudes toward professional services.

### Limitations and Future Directions

The study’s sample size was small and consisted entirely of participants with a minimum graduate-level education. As such, the findings may not reflect the broader experiences of Indian migrants in Australia, particularly those with limited English proficiency, lower educational attainment, or different religious and regional affiliations. As mental health remains a culturally sensitive topic, participants may have underreported personal or family struggles due to stigma or fear of judgment. The reliance on self-reported data also introduces potential bias, particularly with attitudes and service use.

## Data Availability

No datasets were generated or analysed during the current study.

## Appendix 1

**Interview Questions:**

1. Tell me about yourself. Which country did you migrate from originally……,] How long have you been in Australia?

2. Do you have children? What are your thoughts and feelings about being a migrant parent in terms of the mental health and well-being of your children?

3. What is your understanding of having good mental health?

4. Have you had any experience seeking services for mental health in Australia? If so, please describe how the experience was.

5. Are you aware of where to seek help for mental health problems in Australia as a migrant? If so, can you please mention some of these?

6. Do you feel comfortable discussing issues related to mental health with members of your family and your community?

7. In your view, what are some of the barriers to seeking mental health services/support here in Australia?

8. In your view, what are some of the facilitators (what has been helpful) for seeking mental health services/support in Australia?

9. In your opinion, what could be done better when it comes to supporting/promoting good mental health?

10. In your opinion, what could be done better when it comes to supporting/promoting good mental health?
